# Cardiovascular Risk Factors Among Young People in Haiti: Implications for Low-Income Countries

**DOI:** 10.5334/gh.1435

**Published:** 2025-06-10

**Authors:** Lindsey K. Reif, Vanessa Rouzier, Lily D. Yan, Shalom Sabwa, Genevieve Hilaire, Marie Jean Pierre, Rose Cardelle Riche, Robert Peck, Anju Ogyu, Rodney Sufra, Jean W. Pape, Daniel W. Fitzgerald, Margaret L. McNairy

**Affiliations:** 1 Center for Global Health, Department of Medicine, Weill Cornell Medicine, NY, United States; 2 GHESKIO Centers, Port-au-Prince, Haiti; 3 Division of General Internal Medicine, Department of Medicine, Weill Cornell Medicine, NY, United States

**Keywords:** CVD, young adult, youth, hypertension

## Abstract

**Introduction:**

Cardiovascular disease (CVD) is a leading cause of global mortality with >80% of the burden in low-income countries. We investigate population-based estimates of CVD risk factors among young people ages 18–30 in Haiti and provide insights for CVD prevention.

**Methods:**

This is a cross-sectional study within the Haiti Cardiovascular Cohort Study. CVD risk factors include: high blood pressure (BP), dyslipidemia, kidney disease, overweight and obese, and health behaviors. Multivariate logistic regression assessed associated independent factors.

**Results:**

Among 957 participants ages 18–30 years, 23.5% had high BP (95%CI: 20.9%–26.3%), 34.9% had dyslipidemia (95%CI: 31.8%–38.1%), 6.4% had kidney disease (95%CI: 4.8%–8.4%), 16.5% were overweight (95%CI: 14.2%–19.0%), and 6.8% were obese (95%CI: 5.3%–8.6%). More males had high BP (33.6% vs. 14.0%; p < 0.001) and more females had dyslipidemia (45.1% vs. 23.9% p < 0.001). Overweight and obese participants had higher odds of high BP (aOR: 2.05, 95%CI: [1.31–3.19]; aOR 2.15, 95%CI [1.11–4.04]) and dyslipidemia (aOR: 1.70, 95%CI [1.15–2.50]); aOR 2.82, 95%CI [1.63–4.98]) compared to those with normal BMI. Participants ages 25–30 had higher odds of high BP (aOR: 1.58, 95%CI: [1.14–2.18]) and dyslipidemia (aOR: 1.81, 95%CI: [1.35–2.43]) compared to participants ages 18–24.

**Discussion:**

Prevalence of high BP and dyslipidemia are alarmingly high in Haitian young adults, with higher rates of dyslipidemia in women and elevated BP in men. These data provide evidence for routine CVD screening in young people as early as 18 years and underscore the need to identify modifiable drivers of early-onset CVD.

## Introduction

Cardiovascular disease (CVD) is the leading cause of mortality globally, with over 80% of the global CVD burden falling on low-income countries, including Haiti (1). CVD risk factors such as hypertension, dyslipidemia, kidney disease, obesity, tobacco use, and sedentary lifestyle are underlying factors that are correlated with later life cardiovascular events including cardiac dysfunction, heart failure, and mortality (2,3,4). Preceding CVD risk factors is the preclinical phase of CVD, defined as asymptomatic cardiometabolic abnormalities, are associated with the development of premature CVD events and mortality (5,6,7,8,9,10). Our knowledge of the epidemiology of the preclinical phase of CVD among young people in low-income settings is limited. Understanding which preclinical CVD risk factors are most prevalent, the age of onset, and associated independent factors can inform targeted primordial and primary prevention strategies which are essential to prevent the development of disease and ultimately premature CVD. Early intervention—during adolescence and young adulthood—is essential, as studies show later life interventions cannot fully reverse previously acquired CVD risk (7).

Preliminary data from Haiti indicate CVD risk factors emerge earlier in the life-course. For example, hypertension occurs nearly two decades earlier in Haitians than similarly aged Black Americans (11,12). Young people living in Haiti are exposed to a host of structural challenges associated with a low-income setting, including chronic and extreme poverty, food insecurity, and a fragile health infrastructure. These factors may directly influence the development of preclinical CVD risk factors and may intensify exposures that are known to be associated with CVD risk factors such as stress, depression, or poor diet (13,14). Before disentangling multiple, potentially synergistic, risk factors which may contribute to preclinical CVD, we need to establish the epidemiology of CVD among young people in low-income country settings. This includes determining which preclinical CVD risk factors are most prevalent, the age at which these factors emerge, and identifying the independent, modifiable risk factors associated with these conditions. This data will inform screening for young people at the highest risk to help identify and implement prevention interventions.

Adolescence and young adulthood are critical developmental periods when future lifestyle habits and behaviors become engrained and when interventions for primordial CVD prevention can be most effective. Targeted prevention interventions during these periods are supported by the WHO-affiliated World Heart Federation and American Heart Association (AHA) as cost-effective and cost saving (15). Diagnosis of preclinical CVD early in the life-course can trigger interventions that can halt progression, and even reverse course (16). For example, reducing salt intake or increasing physical activity can reduce blood pressure (BP) and prevent hypertension (17,18).

In this study, we report the population-based estimates of CVD risk factors among young people ages 18–30 years in Haiti. This data can provide critical guidance for developing and implementing primordial and primary CVD prevention interventions for young people in low-income countries.

## Methods

### Study Design

This is a cross-sectional analysis within the Haiti CVD Cohort Study, a population-based longitudinal study of 3,005 adults ≥18 years living in Port au Prince, Haiti, who were enrolled between March 2019 and April 2021. Participants in the Haiti CVD Cohort were selected using multistage random sampling using Global Positioning System (GPS) waypoints within census blocks, with the number of waypoints per block proportional to the estimated size of the population. Study procedures have been previously published (https://www.clinicaltrials.gov/study/NCT03892265). The analytic population for this study included all participants ages 18–30 years in this cohort (19).

### Study Setting and Population

This study was conducted at the Groupe Haitian d’etude du Sarcome de Kaposi et des Infections Opportunistes (GHESKIO), which is the largest public health clinic in Haiti, established in 1982 in downtown Port-au-Prince. GHESKIO’s founding mission was the provision of clinical care, research, and training for HIV, and has expanded to include other infectious and chronic diseases including CVD (20).

### Study Procedures and Data Collection

Study procedures have been previously published and described below in brief (19). All data were collected at study enrollment. Sociodemographic data (age, sex, education, and income) were collected using standardized questionnaires. Age was analyzed as a continuous variable and a categorical variable (18–24 years and 25–30 years) to align with the World Health Organization definition of young adult, which is ages 18–24 years. Education was classified as none, primary, or secondary, or higher. Poverty level was defined by income, measured in Haitian Gourdes and categorized as ≤1 US dollar (USD) or >1 USD. Health behaviors including smoking, alcohol intake, and physical activity were collected using standardized World Health Organization STEPwise Approach to NCD Risk Factor Surveillance surveys (21,22). Smoking was categorized as current smoking (yes/no) and lifetime history of smoking (any/none). Alcohol use was categorized as low (≤1 drink per day) or moderate (>1 drink per day). Physical activity was determined using questions about vigorous physical activity and categorized as low (<150 minutes per week) or moderate/high (≥150 minutes per week). Participants completed a physical exam including height and weight (measured using a medical grade mechanical-beam scale with height rod) to calculate BMI (kg/m^2^) and categorized as obese, overweight, normal, or underweight.

BP was measured using the automated Omron HEM-907 machine with an appropriate cuff size (bladder encircling at least 80% of arm), the participant was seated in a quiet space for 5 minutes with both feet on the ground and their arm supported at heart level (23,24). Three unobserved BP measurements were taken on the left arm separated by one-minute intervals with the second and third measurement averaged for analysis.

Laboratory data, including blood and urine, were collected after fasting. Blood specimens were assessed for serum creatinine using a VITROS 250/350 Chemistry System (Ortho Clinical Diagnostics, Raritan, NJ). Urine specimens were assessed for albumin and creatinine using a UniCel DxC 800 Chemistry Analyzer (Beckman Coulter, Brea, CA), with creatinine determined using the Jaffe method (25).

### Study Measures

Preclinical CVD risk factors included high BP, dyslipidemia, and kidney disease. High BP was defined as systolic blood pressure (SBP) >120 mmHg or diastolic blood pressure (DBP) >80 mmHg or a self-report of antihypertensive medication use in the past 2 weeks, per AHA guidelines (22). We further categorized high BP according to AHA guidelines including elevated BP (SBP/DBP ≥120–129/<80), stage 1 hypertension (SBP/DBP ≥130–139/80–89), or stage 2 hypertension (SBP/DBP ≥140/90). Dyslipidemia was defined as low-density lipoprotein cholesterol (LDL-C) ≥100 mg/dL (15). Kidney disease was defined using estimated glomerular filtration rate (eGFR) and urine albumin creatinine ratio (UACR) cutoff valued according to the Kidney Disease Improving Global Outcomes (KDIGO) CKD risk categories. Specifically, kidney disease was defined as a reduced eGFR <60 mL/min/1.73 and/or a UACR ≥30 mg/g, which corresponds to a KDIGO risk of medium of greater (26,27). We report participants with multimorbidity, defined as more than one CVD risk factor including high BP, BMI greater than 25, dyslipidemia, kidney disease, any lifetime tobacco use, moderate or high alcohol use, and physical inactivity.

### Statistical Analyses

Descriptive analyses, including summary statistics, means, medians, interquartile ranges (IQR), and percentages were calculated for sociodemographic variables, health behaviors, and CVD risk factors, and stratified by sex. We conducted univariate and multivariable logistic regression to assess the association between independent factors (age, sex, BMI) and CVD risk factors including high BP and dyslipidemia. All analyses were conducted in R version 4.0.3.

### Ethics

The study protocol and informed consent forms were approved by the Weill Cornell Medicine and GHESKIO institutional review boards (#1803019037). All participants provided written informed consent prior to enrollment in the study.

## Results

### Participant Characteristics

Among 3,005 participants in the Haiti CVD Cohort study, a total of 957 participants were ages 18–30 years and eligible for inclusion in this analysis. Participant median age was 24 years (IQR 21–27), 52% were female, and all participants reported Black race. Most participants (94%) had an education level of secondary school or higher, and 79% reported income of ≤1 USD per day. Socio-demographic characteristics did not significantly differ between male and female participants (Table 1).

**Table 1:** Socio-demographic Characteristics of Young Adult Participants Ages 18–30 Years.


	TOTAL N (%) (N = 957*)	FEMALE N (%) (N = 493)	MALE N (%) (N = 464)	P-VALUE

**Age**				

Median (IQR)	24 (21–27)	25 (21–27)	24 (21–27)	0.60

18–24	494 (51.6)	245 (49.7)	249 (53.7)	0.25

25–30	463 (48.4)	248 (50.3)	215 (46.3)	

**Incom**e				

<1 USD/day	757 (79.5)	381 (77.9)	376 (81.2)	0.24

≥1 USD/day	195 (20.5)	108 (22.1)	87 (18.8)	

**Education**				

None	10 (1.1)	8 (1.6)	2 (0.4)	0.02

Primary	38 (4.0)	26 (5.3)	12 (2.6)	

Secondary or Higher	904 (94.9)	455 (93.1)	449 (97.0)	


*Number of missing data points due to participant non-response include: 5 income; 5 education.

### CVD Risk Factors

The prevalence of high BP was 23.5% (95% CI: 20.9%–26.3%) including 12.1% with elevated BP (95% CI: 10.1%–14.4%), 7.9% with stage 1 hypertension (95% CI: 6.3%–9.8%) and 3.5% with stage 2 hypertension (95% CI: 2.4%–4.8%). Dyslipidemia prevalence was 34.9% (95% CI: 31.8%–38.1%), kidney disease prevalence, defined by medium, high, or very high KDIGO risk category, was 6.4% (95% CI: 5.3%–8.6%) with 6.2% with ACR ≥30 mg/g and 0.21% with eGFR < 60 mL/min/1.73. A total of 16.5% of participants were overweight (95%CI: 14.2%–19.0%) and 6.8% were obese (95%CI: 5.3%–8.6%). Among participants with dyslipidemia (LDL-C > 100 mg/dL), 9.8% of participants were categorized as borderline high or high with an LDL-C > 130 mg/dL (95%CI: 7.9%–11.8%). Other CVD risk factors included 6.3% who reported any lifetime use of tobacco (95%CI: 4.8%–8.0%), 25.6% reported one or more alcoholic drinks per day (95%CI: 22.4%–29.0%), and 46.9% reported low physical activity (95%CI: 43.7%–50.1%). A total of 48.7% of participants had two or more CVD risk factors (Table 2).

**Table 2:** Prevalent CVD Risk Factors among Young Adult Participants Ages 18–30 Years.


	TOTAL N (%) (N = 957*)	FEMALE N (%) (N = 493)	MALE N (%) (N = 464)	p-VALUE

**Blood Pressure**				

Mean Systolic blood pressure (SD)	110.6 (12.6)	106.4 (11.4)	115.2 (12.3)	<0.001

Mean Diastolic blood pressure (SD)	65.0 (10.2)	65.65 (10.2)	64.25 (10.1)	0.05

Normal (SBP < 120 or DBP < 80)	731 (76.5)	423 (85.9)	308 (66.4)	

Elevated BP (SBP 120–129 and DBP < 80)	116 (12.1)	26 (5.3)	90 (19.4)	<0.001

Stage 1 Hypertension (SBP 130–139 or DBP 80–89)	76 (7.9)	27 (5.5)	49 (10.5)	0.005

Stage 2 Hypertension (SBP > 140 or DBP > 90)	33 (3.5)	16 (3.3)	17 (3.7)	0.86

High BP, Stage 1 or 2 Hypertension	225 (23.5)	69 (14.0)	156 (33.6)	<0.001

**BMI**				

Median (IQR)	21.8 (19.8–24.7)	23.2 (20.4–27.0)	20.8 (19.4–23.6)	<0.001

Underweight (BMI < 18.5)	97 (10.2)	38 (7.7)	59 (12.7)	<0.001

Normal (BMI 18.5 – <25)	635 (66.6)	280 (57.0)	355 (76.7)	

Overweight (BMI 25–30)	157 (16.4)	117 (23.8)	40 (8.6)	

Obese (BMI > 30)	65 (6.8)	56 (11.4)	9 (1.9)	

**Dyslipidemia**				

Median (IQR)	88 (71–108)	94 (78–116)	82 (66–99)	<0.001

(LDL-C) ≥ 100 mg/dL	322 (34.9)	216 (45.1)	106 (23.9)	<0.001

(LDL-C) < 100 mg/dL	601 (65.1)	263 (54.9)	338 (76.1)	

**Kidney Disease**				

**Microalbuminuria**				

Urine ACR ≥ 30 mg/g	48 (6.2)	29 (7.6)	19 (4.8)	0.14

Urine ACR < 30 mg/g	727 (93.8)	352 (92.4)	375 (95.2)	

**Creatine clearance**				

eGFR < 60 mL/min/1.73	2 (0.2)	2 (0.4)	0	0.52

eGFR ≥ 60 mL/min/1.73	923 (99.7)	479 (99.6)	444 (100.0)	

KDIGO** risk category (combined eGFR and ACR)				

Low Risk	716 (93.6)	346 (91.2)	369 (95.1)	0.09

Medium Risk/High Risk/Very High Risk	49 (6.4)	31 (8.2)	19 (4.9)	

Diabetes Mellitus	6 (0.01)	3 (0.01)	3 (0.01)	1.0

**Current Smoking Status**				

Current, No	916 (96.3)	475 (97.1)	441 (95.2)	0.23

Current, Yes	35 (3.7)	14 (2.9)	21 (4.5)	

**Lifetime Smoking History**				

Any lifetime smoking	60 (6.3)	17 (3.5)	43 (9.3)	0.001

No lifetime smoking	889 (93.4)	470 (96.5)	419 (90.7)	

**Alcohol Use**				

Low (≤1 drink a day)	521 (74.4)	244 (76.5)	277 (72.7)	0.29

Moderate-high (more than 1 drink a day)	179 (25.6)	75 (23.5)	104 (27.3)	

**Physical Inactivity**				

<150 min/week	446 (46.9)	214 (43.6)	232 (50.1)	0.06

>150 min/week	505 (53.1)	274 (55.8)	231 (49.9)	

**Multiple Risk Factors**				

0 risk factors	160 (16.7)	78 (15.8)	82 (17.7)	0.19

1 risk factor	331 (34.6)	166 (33.7)	165 (35.6)	0.49

2+ risk factors	466 (48.7)	249 (50.5)	217 (46.8)	


*Number of missing data points due to participant non-response or unavailable laboratory results include: 1 BP measurement, 3 BMI, 34 dyslipidemia, 182 microalbuminuria, 32 creatine clearance, 6 current smoking status, 8 lifetime smoking history, 257 alcohol use, 6 physical inactivity, 5 diabetes mellitus.**KDGIO = Kidney Disease Improving Global Outcomes are criteria for diagnosing chronic kidney disease.

### Sex Stratification

**Figure 1:**
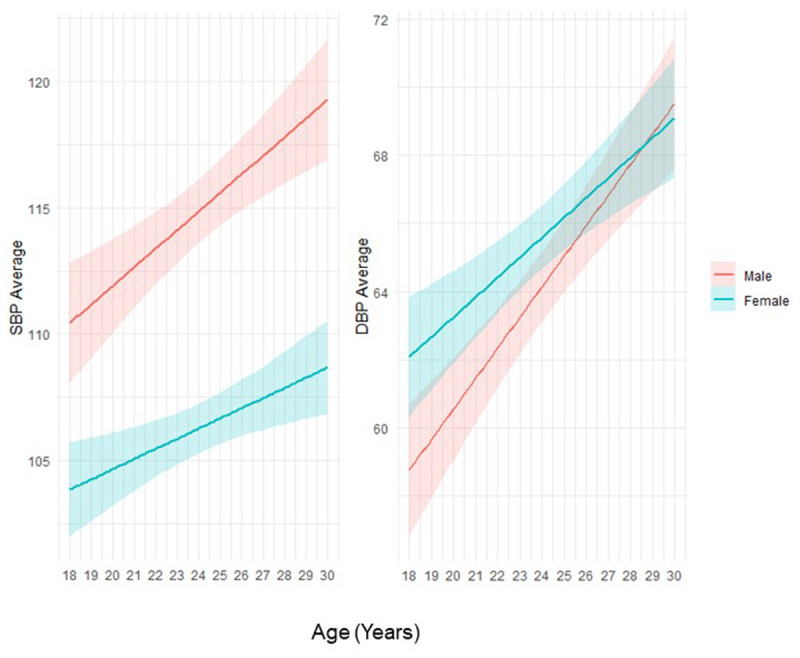
Average Systolic and Diastolic BP by Age, Stratified by Sex.

The prevalence of CVD risk factors varied significantly by sex. Males had a higher prevalence of high BP, with 33.6% compared to 14.0% of females (p < 0.001). Additionally, 9.3% of males reported any lifetime use of tobacco, whereas only 3.5% of females did (p < 0.001). In contrast, females had a higher prevalence of obesity, with 11.4% compared to just 1.9% of males (p < 0.001), as well as a greater prevalence of dyslipidemia, at 45.1% versus 23.9% of males (p < 0.001). Males had significantly higher SBP and females had higher DBP, though this difference was less pronounced. Average SBP and DBP increased with age in both sexes, but at a greater rate among males (Figure 1). A higher proportion of females with dyslipidemia were also categorized as borderline high/high LDL-C–12.9% vs. 6.3% of males (p < 0.001). The proportion of participants with more than one CVD risk factor was high and did not differ by sex. Among all participants, 34.6% had 1 CVD risk factor and 48.7% had 2 or more CVD risk factors, with only 16.7% with no prevalent CVD risk factor (Table 2).

### Associated Factors

Participants ages 25–30 years, compared to 18–24 years, had higher odds of high BP (aOR = 1.58, 95% CI: 1.14–2.18; p = 0.005), and dyslipidemia (aOR = 1.81, 95% CI: 1.35–2.43; p < 0.001), adjusting for sex and BMI. Females had lower odds of high BP (aOR = 0.25, 95% CI: 0.17–0.35; p < 0.001) but higher odds of dyslipidemia (aOR = 2.12, 95% CI: 1.57–2.87; p < 0.001). Compared to normal BMI, being both overweight and obese was associated with higher odds of high BP (aOR = 2.05, 95%CI: 1.31–3.19, p = 0.002 and aOR = 2.21, 95% CI: 1.14–4.04; p = 0.02, respectively) and higher odds of dyslipidemia (aOR 1.70, 95%CI: 1.15–2.50, p = 0.007; aOR = 2.82, 95%CI: 1.63–4.98; p < 0.001, respectively) (Table 3).

**Table 3:** Factors Associated with Elevated Blood Pressure (BP) and Dyslipidemia.


	ELEVATED BP aOR* (95%CI)	p-VALUE	DYSLIPIDEMIA aOR* (95%CI)	p-VALUE

**Age**				

Per 1-year increase	1.10 (1.06–1.15)	<0.001	1.13 (1.08–1.17)	<0.001

18–24 years	1 (ref)		1 (ref)	

25–30 years	1.52 (1.09, 2.11)	0.005	1.81 (1.34–2.44)	<0.001

**Sex**				

Male	1 (ref)		1 (ref)	

Female	0.23 (0.16, 0.33)	<0.001	2.13 (1.57–2.89)	<0.001

**BMI**				

Normal (BMI 18.5 – < 25)	1 (ref)		1 (ref)	

Underweight (BMI < 18.5)	0.85 (0.48, 1.45)	0.572	0.57 (0.32–0.98)	0.05

Overweight (BMI 25–30)	2.21 (1.41, 3.47)	0.002	1.73 (1.17–2.55)	0.007

Obese (BMI > 30)	2.44 (1.25, 4.64)	0.020	2.80 (1.61–4.96)	<0.001

**Income**				

<1 USD/day	1 (ref)		1 (ref)	

≥1 USD/day	0.90 (0.60–1.32)	0.59	0.99 (0.69–1.41)	0.96

**Education**				

None	3.85 (0.91–14.77)	0.05	1.46 (0.39–5.52)	0.56

Primary	1.41 (0.62–3.00)	0.38	0.68 (0.31–1.41)	0.31

Secondary or Higher	1 (ref)		1 (ref)	


*Model adjusted for age, sex, BMI, income, and education.

## Discussion

These data provide previously unknown estimates of early-onset CVD risk factors among young adults in a setting of extreme poverty and adversity. Rates of high BP and dyslipidemia are alarmingly high for this group of young adults so early in their life course. Over one third of young men and 1 in 10 young women had prevalent high BP. Young women had a particularly high prevalence of dyslipidemia at almost 1 in 2 compared to 1 in 5 young men. These early onset CVD risk factors are likely predictive of CVD events later in adulthood, given high BP is predictive of incident hypertension (28) and dyslipidemia is predictive of atherosclerotic disease (29,30,31). In turn, these risk factors are correlated with grave CVD outcomes including heart failure, ischemic heart disease, stroke, and mortality (2,3,32). These data provide evidence to support routine screening for high BP and dyslipidemia of young people as early as age 18 years and underscore the need for additional research to identify the onset of preclinical markers even before young adulthood, and their associated poverty-related social determinants.

Hypertension is a well-established risk factor for the development of heart disease including heart failure and stroke (28). The prevalence of hypertension in the US National Health and Nutrition Examination Surveys (NHANES) 2017–2020 data, among participants ages 20–44 years, was similar to our findings. Given our younger age range of 18–30 years, this is concerning and indicates potentially an earlier onset of elevated BP and hypertension among Haitians. In the US National Longitudinal Study of Adolescent to Adult Health study, which followed young people ages 11–18 years through young adulthood ages 24–32 years, male sex and Black race/ethnicity were both associated with higher odds of hypertension compared to females and White race/ethnicity, respectively (33). Potential drivers of earlier onset of high BP in our population may include poverty-specific social and environmental determinants in a younger population. For example, another analysis from the Haiti CVD Cohort Study found that neighborhoods with the most extreme social vulnerabilities (e.g., poverty, food insecurity, neighborhood violence, etc.) as compared to neighborhoods with lesser vulnerabilities had higher association of hypertension, with the association greatest among young people <45 years (34). Studies on BP trajectories across the life-course indicate that persistent, sustained high BP from young adulthood through middle adulthood results in adverse cardiovascular outcomes in middle age (35). Further research is needed to understand the acceleration of high BP to hypertension in this population but regardless, this data underscores the need for early screening and primary prevention in young adulthood in Haiti. A quarter of our study population had high BP and would be a candidate for early intervention.

Dyslipidemia rates in our study were similar to those found in US populations in NHANES 2003 to 2020 (36). Approximately one third of Haitian young adults ages 18–30 years in our study had dyslipidemia compared to over half of US young adults ages 20–39 years. However, among the US population in NHANES, a significantly greater proportion of men had dyslipidemia compared to women. In our study, young adult females were twice as likely to have dyslipidemia and more likely to be overweight or obese. Dyslipidemia was defined as LDL-C level of >100 mg/dL, consistent with definitions from U.S.-based longitudinal cohort studies indicating that even moderately elevated LDL-C levels in young adulthood are associated with increased CVD risk later in life (37,38). This lower threshold prioritizes early identification of risk and may capture individuals who would not be classified with dyslipidemia under European guidelines, which define elevated LDL-C as >116 mg/dL (39). As a result, our dyslipidemia prevalence estimates may be higher, reflecting a more preventive approach to cardiovascular risk stratification in young adults. Data on dyslipidemia among young people in low-income countries is scarce, and additional drivers behind the risk for early dyslipidemia among young Haitian women needs to be explored.

Studies among young adults in the US have shown that exposure to elevated LDL-C between ages 18–40 years is significantly associated with risk of incident CVD events. Furthermore, higher LDL-C between the ages of 18–30 increased later life CVD risk more than low LDL-C during that same age range and high LDL-C between the ages 30–40. This indicates that early exposure to higher LDL-C, before the age of 30, cannot be reversed, making early life primordial and primary prevention essential to prevent increased risk for CVD events after the age of 40 (7).

These data highlight the need for optimizing CVD screening and prevention interventions for young people in urban Haiti, which may reflect similar needs in other low-income global settings. Traditional CVD risk assessment typically does not start until age 40, and risk scores for young adults are lacking. Uptake of CVD prevention interventions among young people can be challenging due to a combination of factors: lack of symptoms and low levels of perceived personal risk mean it may be hard to engage young people who feel healthy or do not present for routine clinical care (40). Interventions must be tailored to address these potential barriers to meet young people’s preferences for health services. This could include offering services in a community- or school-based environment, adapting counseling messages so that they are appropriate for young people’s developmental and health literacy levels, and targeting modifiable risk behaviors (e.g., high salt diet) with feasible strategies for behavior change (e.g., how to influence household meals). Providers need to be trained in youth-friendly approaches to CVD screening and prevention to ensure optimal, high-quality provision of services and increase retention of young people.

Strengths of this study include that it is the only population-based study design focused on CVD risk factors in the region. Further, our methods include the use of WHO and AHA blood pressure measurement procedures and inclusion of disaggregated data for young adults under the age of 30. Limitations include potential lack of generalizability to cohorts in rural settings and the lack of home-based or 24-hour ambulatory blood pressure measurements to confirm high BP. Other limitations include the cross-sectional study design precluding the ability to assess temporal relationships between time-varying independent variables and CVD risk factors, a sample size limited by age, given our focus on young adults within the larger population-based cohort which may limit generalizability to the general population in Haiti and other settings.

## Conclusion

Young adults in Haiti have high rates of early-onset high BP and dyslipidemia underscoring the need for targeted lifestyle and developmentally appropriate behavioral interventions for CVD prevention and treatment.
